# Identification and quantification of phytochelatins in roots of rice to long-term exposure: evidence of individual role on arsenic accumulation and translocation

**DOI:** 10.1093/jxb/eru018

**Published:** 2014-03-05

**Authors:** Bruno Lemos Batista, Meher Nigar, Adrien Mestrot, Bruno Alves Rocha, Fernando Barbosa Júnior, Adam H. Price, Andrea Raab, Jörg Feldmann

**Affiliations:** ^1^Centro de Ciências Naturais e Humanas, Universidade Federal do ABC, Bloco B, Av. dos Estados 5001, Santo André (SP), Brazil; ^2^Faculdade de Ciências Farmacêuticas de Ribeirão Preto, Universidade de São Paulo, Bloco A, Av. do Café s/n, Ribeirão Preto (SP), Brazil; ^3^Institute of Biological and Environmental Sciences, University of Aberdeen, Cruickshank Building, St Machar Drive, Aberdeen AB24 3UU, Scotland, UK; ^4^Soil Science Group, Institute of Geography, University of Bern, Hallerstrasse 12, 3012 Bern, Switzerland; ^5^Departamento de Química, Faculdade de Filosofia Ciências e Letras de Ribeirão Preto, Universidade de São Paulo, Avenida Bandeirantes, 3900, Ribeirão Preto (SP), Brazil; ^6^TESLA (Trace Element Speciation Laboratory), Department of Chemistry, University of Aberdeen, Meston Walk, Aberdeen AB24 3UE, Scotland, UK

**Keywords:** Arsenic transfer factor, arsenic uptake, HPLC-ICP-MS/ESI-MS, phytochelatins, rice

## Abstract

Rice has the predilection to take up arsenic in the form of methylated arsenic (o-As) and inorganic arsenic species (i-As). Plants defend themselves using i-As efflux systems and the production of phytochelatins (PCs) to complex i-As. Our study focused on the identification and quantification of phytochelatins by HPLC-ICP-MS/ESI-MS, relating them to the several variables linked to As exposure. GSH, 11 PCs, and As–PC complexes from the roots of six rice cultivars (Italica Carolina, Dom Sofid, 9524, Kitrana 508, YRL-1, and Lemont) exposed to low and high levels of i-As were compared with total, i-As, and o-As in roots, shoots, and grains. Only Dom Sofid, Kitrana 508, and 9524 were found to produce higher levels of PCs even when exposed to low levels of As. PCs were only correlated to i-As in the roots (*r*=0.884, *P* <0.001). However, significant negative correlations to As transfer factors (TF) roots–grains (*r*= –0.739, *P* <0.05) and shoots–grains (*r*= –0.541, *P* <0.05), suggested that these peptides help in trapping i-As but not o-As in the roots, reducing grains’ i-As. Italica Carolina reduced i-As in grains after high exposure, where some specific PCs had a special role in this reduction. In Lemont, exposure to elevated levels of i-As did not result in higher i-As levels in the grains and there were no significant increases in PCs or thiols. Finally, the high production of PCs in Kitrana 508 and Dom Sofid in response to high As treatment did not relate to a reduction of i-As in grains, suggesting that other mechanisms such as As–PC release and transport seems to be important in determining grain As in these cultivars.

## Summary

Six varieties of rice were exposed to low and high levels of arsenic in the same soil. Their individual responses of expressing phytochelatins have been correlated to inorganic arsenic uptake, transport, and accumulation in the rice grain.

## Introduction

Rice (*Oryza sativa* L.) is a global staple food which needs monitoring for contaminants. In a market basket survey in the United States, rice was found to have higher concentrations of inorganic arsenic (i-As) than other commodities (Schoof *et al.*, 1999). Other studies demonstrated that this cereal accumulates more arsenic (As) in shoots and grains than wheat and barley (Williams *et al.*, 2007). Rice consumption may therefore represent a significant risk, mainly when As is present in its inorganic forms, arsenite (As^III^) and arsenate (As^V^), which are class 1 non-threshold carcinogens (ATSDR, 2007; Meharg *et al.*, 2009).

Arsenic uptake by rice plants depends on several factors. Cultivation under flooded conditions (Xu *et al.*, 2008), irrigation with As contaminated water, as well as soil naturally contaminated with As all increase the levels of this element in rice grains (Saha and Ali, 2007; Lu *et al.*, 2009; Meharg *et al.*, 2009). Organic As (o-As) species (dimethylarsinic acid (DMA) and monomethylarsonic acid (MMA)) are absorbed to a lesser extent than i-As (Raab *et al.* 2007b). As^III^ and As^V^ are mostly carried via silicate and phosphate transporters, respectively, probably due to molecular similarities with silica and phosphate (Meharg *et al.* 1994; Ma *et al.*, 2006; Zhao *et al.*, 2010). After entering the root cells, As^V^ is readily converted to As^III^ and the Lsi1 efflux channels can expel As^III^ out of the root or into the xylem (via Lsi2), leading to transport into shoots and grains (Ma *et al.*, 2006; Zhao *et al.*, 2010). Another defence system based on binding i-As in roots is the formation of As–phytochelatin complexes and their sequestration into cell vacuoles (Raab *et al.*, 2005, 2007*a*; Song *et al.*, 2010). The ability to synthesize phytochelatins (PCs) depends on genetic factors and the availability of sulphate (Cobbett and Goldsbrough, 2002; Zhao *et al.*, 2010; Duan *et al.*, 2011).

PCs are derived from glutathione (GSH). Zhang *et al.* (2010) and Duan *et al.* (2011) observed that sulphur and GSH/PC deprivation increased As translocation in rice from roots to shoots and from shoots to grains, respectively. Duan *et al.* (2011) reported diverse patterns of As and PC accumulation in rice plants from different cultivars. They concluded that PCs have a specific role in As translocation and As accumulation in rice grains, without identification or quantification of the involved thiols.

The aim of this study was to compare PC production and As–PC complex formation in six different rice cultivars exposed to long-term high and low As levels in order to study their influence on the As translocation in plants. The use of high performance liquid chromatography (HPLC) hyphenated simultaneously to inductively coupled plasma mass spectrometry (ICP-MS) and electrospray mass spectrometry (ESI-MS) allowed simultaneous identification and quantification of As–peptide complexes and peptides without the need for authentic standards. Furthermore, the potential of these thiol compounds to serve as biomarkers for As exposure and their relationship to As translocation in rice were evaluated.

## Materials and methods

### Reagents

Ultra-pure water (Elga Ltd., High Wycombe, Bucks, UK) was used throughout the experiments. All reagents used were of analytical grade. Formic acid (98%), nitric acid (67%), and hydrogen peroxide (30%) were purchased from Fluka (UK). Potassium phosphate dibasic (99%), di-sodium arsenate (Na_2_HAsO_4_.7H_2_O) and *N*-acetyl-cysteine (99%) were purchased from Sigma–Aldrich. MMA and DMA were purchased from Strem Chemicals (Newburyport, USA). Elemental standard solutions (Ge, Ga, and As, 1000mg l^–1^) were obtained from High Purity Standards (Charleston, USA) and methanol from Fisher (London, UK).

### Instruments

The instruments used for speciation were an Accela HPLC system coupled (split 1:4) with an ICP-MS Element 2 and an ESI-MS LTQ Orbitrap Discovery (Thermo Fisher Scientific, Germany) for the determination of PCs and As–PC complexes. For As species determinations in grains, an 1100 HPLC system coupled with an 7500c ICP-MS (Agilent Technologies Stockport, Cheshire, UK) was used. Total As analysis was performed using an ICP-MS Agilent 7500c.

### Rice cultivation conditions

Six different rice cultivars (*Oryza sativa* L.), Italica Carolina (IC), 9524 (9), Lemont (L), Kitrana 508 (K), Dom-Sofid (DS), and YRL-1 (Y) were selected from the Rice Diversity Panel 1 (Zhao *et al.*, 2011). The rice lines were selected based on a study of grain arsenic in over 300 cultivars grown in the field in Bangladesh and China (Norton *et al.*, 2012) where Lemont was low in both, Dom Sofid and Italica Carolina were low in Bangladesh but high in China, 9524 and Kitrana 508 were high in Bangladesh and low in China, and YRL-1 was high in both. In addition, in Bangladesh, 9524 and YRL-1 were identified as having a high shoot to grain As transfer factor while Dom Sofid and Italica Carolina were low.

After germination, plants were individually potted (eight plants each) and grown under flooded conditions (2.0 l-pots, soil immersion under 3–4cm of water) and greenhouse conditions. The soil used for the experiment contained an average of 7.5mg As kg^–1^ at the outset (low exposure level). Half of the plants from each cultivar received, after transplanting, 10mg As in the form of As^V^ per pot (high exposure level). During growth the plants were regularly fertilized using Yoshida’s nutrient solution (Yoshida *et al.*, 1976) and grown until the grains were mature without additional exposure to As. Just before harvesting, plant height, grain length (with husk), and the internodes stem diameter (mean of smaller diameter of the first internodes of all stems from a single plant) were measured by using a caliper ruler.

### Sample preparation

Shoot lengths and all plant weights were determined after harvesting. Soil from each pot was collected and the individual plant parts (grains, shoots, and roots after removing the soil) were weighed. A portion of fresh roots (~25g) were washed with tap water for 10min and placed in 100mM phosphate solution (Meharg and MacNair, 1992) to remove any surface-bound As.

Fresh root materials were used for the determination of the As–PC complexes and thiol compounds present. Sample preparation followed the procedure described in Raab *et al.* (2004) and Bluemlein *et al.* (2008). Briefly, roots were ground in liquid nitrogen and extracted on ice for 1h with 1% (v/v) formic acid. After centrifugation, the supernatant was immediately injected into the HPLC-ICP-MS/ESI-MS. This method has been shown in plants to guarantee the integrity of the As–PC complexes and to prevent *de novo* synthesis of As–PC complexes during extraction and analysis (Bluemlein *et al.*, 2008, 2009). The standards of DMA and N-acetyl-cysteine were daily prepared for the quantification of As and sulphur, respectively.

Plants and soil were individually ground and oven-dried at 50 ^o^C for the determination of dry weight, total As (t-As), and speciation of i-As and o-As in grains. Samples for total As were digested with nitric acid/hydrogen peroxide in an open microwave system (MARS5, CEM). Three reference materials (NIST SRM 1568a, IAEA 140 TM, and NCZ2C73007, rice grains, algae, and soil, respectively) were used for quality control purposes. For i-As and o-As determination, grains were extracted following the procedure of Sun *et al.* (2009). Briefly, ground grains were extracted with 1% (v/v) nitric acid using the microwave system above. The supernatant was mixed 1:5 v/v with hydrogen peroxide and stored overnight for complete oxidation of As^III^ to As^V^ (Hansen *et al.*, 2010). Then the samples were injected into a HPLC-ICP-MS.

### Conditions used during speciation

#### As-PCs and thiol-compounds. 

These compounds were separated using a reversed-phase column (Eclipse, XDB-C18, 5 µm, 150×4.6mm, Agilent) with a linear water/methanol (0.1% formic acid) gradient from 0–20% methanol (Accela HPLC system). The column was kept at 30 °C, the autosampler compartment at 4 °C and 0.1ml sample was injected. The column effluent was split with one part going into the ICP-MS (Element 2) mixed via a T-piece with Ga as the internal standard. The ICP-MS was used in medium resolution with nickel cones and a PFA micro-nebulizer. The instrument was optimized using standard conditions. The rest of the column effluent was directed into an OrbiTrap ESI-MS optimized daily and calibrated using standard conditions, and used in positive mode (capillary voltage 4.5kV) with 30 000 resolution. MS^2^ spectra were recorded when the signal intensity was above 50 000 counts. Then the influence of the gradient on the ICP-MS signal was calculated daily according to the procedure published by Amayo *et al.* (2011). Compounds were identified from the mass spectra and quantified using *N*-acetyl-cysteine and DMA, respectively (see Supplementary Table S3 available at *JXB* online).

#### Inorganic and methylated As. 

The separation of i-As and o-As in grains was performed using a Hamilton PRP X-100 column (10 μm, 150×4.1mm) and the column oven set to 30 °C. Mobile phase (1ml min^–1^ flow rate) consisted of 6.66mM NH_4_H_2_PO_4_ and 6.66mM NH_4_NO_3_ at pH 6.2 (Williams *et al.*, 2005). Sample volume injected was 50 µl. The outlet of the HPLC (Agilent 1100) was connected to the ICP-MS (Agilent 7500c) via a T-piece used for mixing the internal standard (Ga 10 µg l^–1^) into the column effluent. The ICP-MS was optimized daily for maximum sensitivity and used in normal mode. DMA was used for calibration and quantification was done based on peak areas. DMA, As^V^ and MMA standard solutions were used for the identification of species (retention time).

### Arsenic transfer factor evaluation

The soil/roots, soil/shoots, soil/grains, roots/shoots, roots/grains, and shoots/grains As transfer factors (TFs) were calculated following the procedure published by Raab *et al.* (2005), where the total As in the plant part of destination is divided by the total As in the origin sample.

### Statistical analysis

The results were analysed by one-way ANOVA followed by Duncan’s multiple range test and critical ranges and correlations were tested by Pearson. Statistica (V 6.0) and SigmaStat (V 3.5) statistical software packages were used. The confidence interval used was set to higher than 95%.

## Results

### Total As concentration and agronomic parameters

**Fig. 1.**
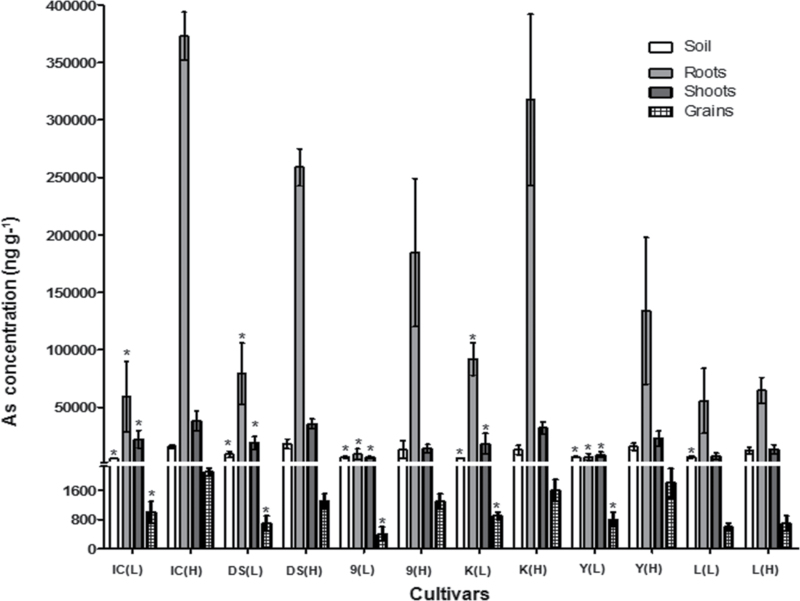
Total arsenic concentration on a dry weight basis in soil and rice tissue from six cultivars exposed to low and high arsenic concentrations. Results presented as mean ±standard deviation; *n*=4; IC, Italica Carolina; DS, Dom Sofid; 9, 9524; K, Kitrana 508; Y, YRL-1; L, Lemont; (L), low exposure; (H), high exposure; *, statistical differences between low and high (*P* <0.05).

The recovery of certified reference materials varied between 49% for soil and 105% for plant materials. Soil As concentrations were 7.0 and 14.9mg kg^–1^ for low and high exposure levels, respectively (Fig. 1). The soil As availability to plants depends on factors such as mineral composition (Fe, P), organic matter content, redox potential and others (Tu and Ma, 2003; Cao and Ma, 2004). Since these factors were similar for all plants, it was enough to confirm at which level the different groups were exposed to As. The high exposure level was achieved by spiking the soil used for low exposure levels with As^V^. At low exposure levels, total arsenic (t-As) in roots varied from 6.9mg kg^–1^ in YRL-1 to 92.1mg kg^–1^ in Kitrana 508, in shoots from 6.7mg kg^–1^ in 9524 to 22.3mg kg^–1^ in Italica Carolina, and in grains from 0.4mg kg^–1^ in 9524 to 1.0mg kg^–1^ in Italica Carolina (Fig. 1). At high exposure levels, Lemont and Italica Carolina presented the lowest and highest concentrations, respectively (roots: 64.8 and 373.0mg kg^–1^; shoots: 13.5 and 38.2mg kg^–1^; grains: 0.7 and 2.1mg kg^–1^, respectively; Fig. 1). The comparison between low and high exposure samples per cultivar showed significant differences (*P* <0.05) between t-As concentrations in roots, shoots, and grains in five cultivars. Lemont did not show a significant difference upon exposure to different As levels in soil (Fig. 1).

Generally, all plants per cultivar exposed to high As levels or not, flowered at the same time and the number of shoots were not different between low and high exposure plants (see Supplementary Table S1 available at *JXB* online). High As concentrations in soil significantly reduced plant height only for Italica Carolina (–11.7%) which also showed a reduced total plant mass. Lemont was the only cultivar where the shoot diameters were reduced (–21.4%) (see Supplementary Table S1 available at *JXB* online). High As treatment reduced grain yield by 13.9–54.7% in all six cultivars. Exposure to elevated As reduced the grain size for Italica Carolina, 9524, and Kitrana 508 (see Supplementary Table S1 available available at *JXB* online).

Positive strong correlations were observed between all agronomic parameters (flowering, plant height, shoots’ diameters, weight of roots, shoots, and whole plant). Grain weights were strongly correlated with the weight of roots, shoots, and, consequently, the whole plant (see Supplementary Table S2 available at *JXB* online). Total As influenced all these parameters. Significant negative correlations were found between (i) t-As in: soil, roots or shoots versus grain weights; and (ii) t-As in shoots versus stem diameters, weight of roots, shoots, and grains (see Supplementary Table S2 available at *JXB* online).

### Arsenic transfer factors

**Fig. 2.**
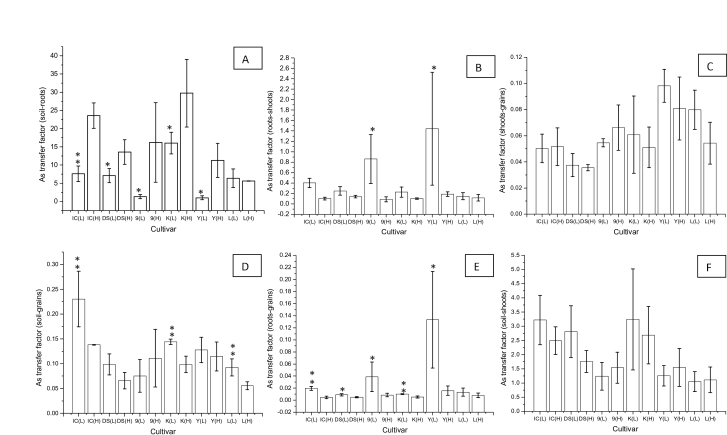
Arsenic transfer factors between soil and plant parts from the cultivars studied. Results presented as mean ±standard deviation. IC, Italica Carolina; DS, Dom Sofid; 9, 9524; K, Kitrana 508; Y, YRL-1; L, Lemont; (L), low exposure; (H), high exposure; statistical differences between low and high exposure samples, *P <0.05; **P <0.01.

The TF is the measurement of the plant’s potential to take up a specific element/compound from soil, transferring it to roots, shoots and grains (Carbonell *et al.*, 1998; Raab *et al.*, 2007b). All cultivars, except Lemont, showed significant difference in TF_soil–root_ depending on As exposure, varying between 2.7 and 28.5 (Fig. 2A). TF_root–shoot_ were only significantly different for 9524 and YRL-1 (Fig. 2B). YRL-1 had the highest TF_root–shoot_ compared with other cultivars (Fig. 2B). As exposure did not significantly affect TF_shoot–grain_ ratios (*P* >0.05) to any cultivar (Fig. 2C). High exposure to As significantly reduced TFs_soil–grain_ for Lemont and Kitrana 508 (Fig. 2D). Exposure to elevated As reduced TFs_root–grain_ for all cultivars (Fig. 2E), whereas TF_soil–shoot_ were independent of exposure level (Fig. 2F).

### Arsenic species in grains

The species identified in grains were i-As as As^V^, MMA, and DMA. No cationic species such as tetramethylarsonium, as found by Hansen *et al.* (2010), were present in grains. NIST 1568a rice flour was used as the reference material for extraction efficiency (~85%) and species quantification, which was similar to other studies reported in the literature (Batista *et al.*, 2011).

**Fig. 3.**
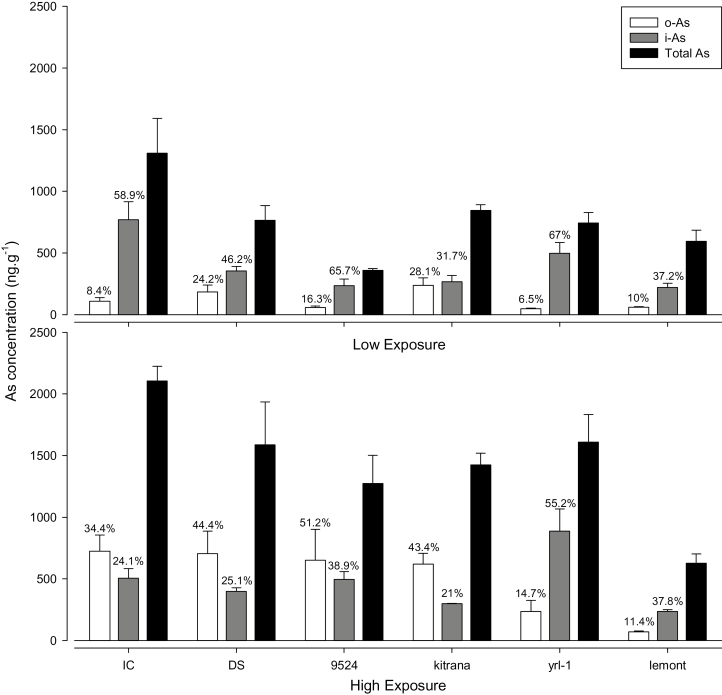
Total As concentration, inorganic As (i-As), and organic As (MMA+DMA, o-As) in grains of rice cultivars exposed at low (L) and high (H) As levels (dry weight). The percentage of o-As and i-As in relation to total arsenic (not sum of species) is above the bars.

Regarding the low exposure level, i-As concentrations ranged from 221ng g^–1^ for Lemont to 772ng g^–1^ for Italica Carolina and o-As from 49ng g^–1^ for YRL-1 to 238ng g^–1^ for Kitrana 508. Under these conditions, the main specie found in grains was i-As for all cultivars (Fig. 3). By contrast, exposure to high As levels increased the amount of t-As (Fig. 1) mainly due to o-As species in grains (Fig. 3). In this case, i-As levels ranged from 237ng g^–1^ for Lemont to 888ng g^–1^ for YRL-1 and o-As from 71ng g^–1^ for Lemont to 725ng g^–1^ for Italica Carolina. Considering all the experiments, t-As, i-As, and o-As were positively correlated with each other (*P* <0.05).

### As–PC complex and thiol compound concentrations

**Fig. 4.**
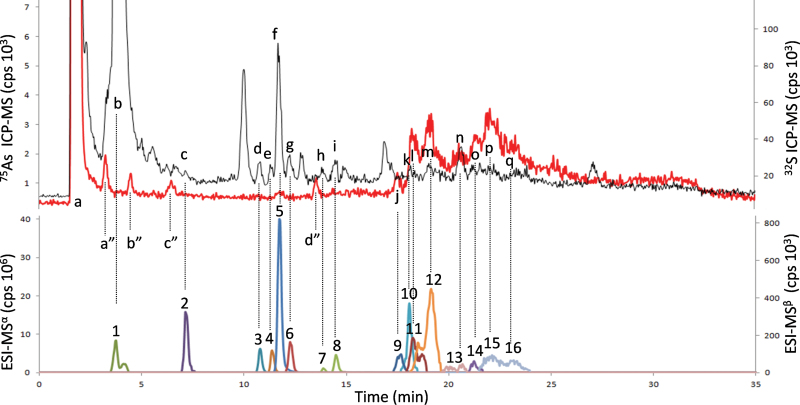
Determination by HPLC/HR-ICP-MS/LTQ Orbitrap ES-MS of PCs and PCs-As in roots of the Italica Carolina cultivar highly exposed to arsenate. HR-ICP-MS, ^32^S (black line); ^75^As (red line). LTQ Orbitrap ES-MS: 1^α^, GSH; 2^β^, GSSG; 3^α^, OH-Me-PC_2_; 4^α^, PC_2_ reduced; 5^α^, DesGly-PC_2_; 6^α^, PC_2_ auto-oxidized; 7–8^α^, Iso-PC_2_-Glu; 9^β^, OH-Me-PC_3_-As; 10^β^, PC_3_; 11^β^, PC_3_-As; 12^β^, DesGly-PC_3_-As; 13^β^, Iso-PC_3_-Glu; 14^β^, Iso-PC_3_-Glu-As; 15–16^β^, PC_4_-As. a, peaks of inorganic As and sulphate; j, l, m, o, p, q, peaks of PCs-As; a′′, b′′, c′′, d′′, As-complexes unidentified; b–q, peptides containing S or As and the corresponding LTQ Orbitrap ES-MS signals.

Rice, similar to most grasses, produces a variety of PCs. By using MS and MS^2^ data members of the following groups were identified: PCs, γ(GluCys)_n_Gly) *n*=2–4; Ser–PCs, γ(GluCys)_n_Ser *n*=2–4; Des–PCs, γ(GluCys)_n_ *n*=2–3; and Glu–PCs, γ(GluCys)_2_Glu *n*=2–3. Roots exposed to high As levels also contained, in addition to free-PCs, a variety of As–PC complexes in the extract. Dominant among these were As–PC_3_, As–PC_4_, As–Des–PC_3_, EC–As–EC–PC_2_, As–(Des–PC_2_)_2_, As–Des–PC_4_, As–Ser–PC_3_, As–Ser–PC_4_, and As–Glu–PC_3_. The mixture of PCs such as (γ-GluCys)_n_Glu, (γ-GluCys)_n_Gly, (γ-GluCys)_n_Ser, and (γ-GluCys)_n_ is characteristic of the Poaceae family (grasses) (Klapheck *et al.*, 1994; Cobbett and Goldsbrough, 2002) (see Supplementary Table S3 available at *JXB* online; Fig. 4). Non-complexed As was the dominant form of As in roots independent of exposure level and rice cultivar. In roots exposed to low levels of As, between 0% and 27% of t-As was complexed by PCs. On the other hand, at high As exposure levels, this increased from 45 to 38%, depending on cultivar. The particular action of phytochelatins on As transport and storage explains this slight increase which is discussed in the next section.

GSH and its oxidized form GSSG were the dominant thiol-containing peptides in all analysis (Table 1). GSH concentration was reduced at high As exposure in Kitrana 508, Dom Sofid, YRL-1, and Lemont. PCs were expressed by all plants. Dom Sofid produced the highest amount of PCs when compared with other cultivars.

**Table 1.** Concentration of GSH, GSSG, and As–PCs complexes found in fresh roots from six different rice cultivarsResults expressed as nmol PC kg^–1^ of fresh roots ±standard deviation; *statistical differences between low (L), *n*=4 and high (H), *n*=4 exposure levels (*P* <0.05); ND, not detected; IC, Italica Carolina; DS, Dom Sofid; 9, 9524; K, Kitrana 508; Y, YRL-1; L, Lemont.

Cultivar	[GSH]	[GSSG]	[OH–Me–PC_2_]	[PC_2_Red]	[DesGly–PC_2_]	[PC_2_Oxi]	[Iso–PC_2_–Glu]
**IC(L)**	109±69*	10±7.7	2±0.2*	3±0.7*	3±0.6*	4±1.0*	7±1.5*
**IC(H)**	1452±201	6±3.4	5±1.2	6±2.7	21±9.3	9±3.7	13±4.0
**DS(L)**	2914±1671	26±9.3	44±16.2	40±21	33±13*	27±8.4*	58±16.4
**DS(H)**	625±573	32±19.1	32±6.3	42±16	50±12	42±3.5	73±24.4
**9(L)**	137±52*	8±6.7	4±1.7	5±1.9	5±2.4	5±2.2	3±1.7*
**9(H)**	1254±466	5±2.5	4±2.2	5±2.2	8±3.5	5±2.1	10±3.6
**K(L)**	944±495*	8±2.4	5±1.6	6±2.7	13±2.8*	9±3.1	16±2.1*
**K(H)**	174±78	8±2.9	6±2.5	8±0.9	23±6.1	13±5.1	24±3.4
**Y(L)**	2296±2198	5±2.2	3±0.7	3±1.0	6±3.9	5±2.3	7±4.8
**Y(H)**	733±123	4±0.9	3±0.6	2±0.9	5±1.8	2±0.4	5±1.3
**L(L)**	1325±1101	9±6.0	4±1.5	5±1.4	7±0.6	6±1.1	13±5.1
**L(H)**	355±192	5±1.2	6±0.9	5±1.1	7±0.6	6±1.5	9±2.1
**Cultivar**	**[PC_3_]**	**[As-PC_3_]**	**[As-Iso-PC_3_-Glu]**	**[As-DesGly-PC_3_]**	**[As-PC4]**	**[Iso-PC_3_-Glu]**	**[As-OH-Me-PC_3_]**
**IC(L)**	2±0.8*	ND	ND	ND	ND	2±0.5*	ND
**IC(H)**	5±0.6	0.2±0.1	0.2±0.03	0.4±0.3	1.4±0.7	12±5.0	0.2±0.1
**DS(L)**	27±16.7	3.7±2.2	3.7±2.3	3.8±1.6*	12.1±5.1*	27±11.7*	2.4±1.5
**DS(H)**	28±4.5	5.3±2.4	4.4±3.4	16.0±7.4	24.3±8.4	74±17.2	2.6±1.0
**9(L)**	2±0.6*	ND	ND	ND	ND	ND	ND
**9(H)**	4±1.2	5.2±1.9	ND	6.1±3.5	26.0±5.9	3±1.6	6.2±5.4
**K(L)**	8±3.8	3.0±1.4	ND	8.9±3.5	18.0±6.0	13±6.0*	2.4±2.3
**K(H)**	14±12.3	4.9±2.4	9.0±5.4	17.4±7.3	23.9±11.0	46±26.2	7.2±10.4
**Y(L)**	1±0.8	ND	ND	ND	ND	ND	ND
**Y(H)**	2±0.5	1.5±0.6	1.3±0.1	3.7±2.7	13.2±6.4	3±1.6	1.3±0.1
**L(L)**	1±0.5	ND	ND	ND	ND	ND	ND
**L(H)**	1±0.6	2.8±1.4	ND	5.2±2.7	14.5±6.3	ND	2.1±0.6

Different As levels in soil did not have a significant influence on PCs concentration in YRL-1 (high grain As accumulator and medium TF_root–grain_) and Lemont (low grain As accumulator and low TF_root-gain_) (Figs 1, 2), showing that PCs are important for some cultivars while other varieties seem to have different mechanisms for As detoxification.

### Correlations between As–PCs complex, thiol compounds, arsenic species, and translocation factors of arsenic

Total As levels in roots were positively correlated with some non-complexed PCs for all cultivars to low exposure (*n*=24 where DesGly–PC_2_: *r*=0.648, *P* <0.01; PC_2_oxi, *r*=0.535, *P* <0.01; Glu–PC_2_, *r*=0.691, *P* <0.01, and PC_3_, *r*=0.514, *P* <0.05). Considering high exposure situations, the correlations were weaker (*n*=24 where DesGly–PC_2_, *r*=0.480, *P* <0.05; PC_2_oxi, *r*=0.365, *P* <0.05; Glu–PC_2_, *r*=0.273; *P* >0.05; and PC_3_, *r*=0.364, *P* <0.05). By contrast, As–PC complexes and other thiol compounds did not correlate with t-As in roots.

**Fig. 5.**
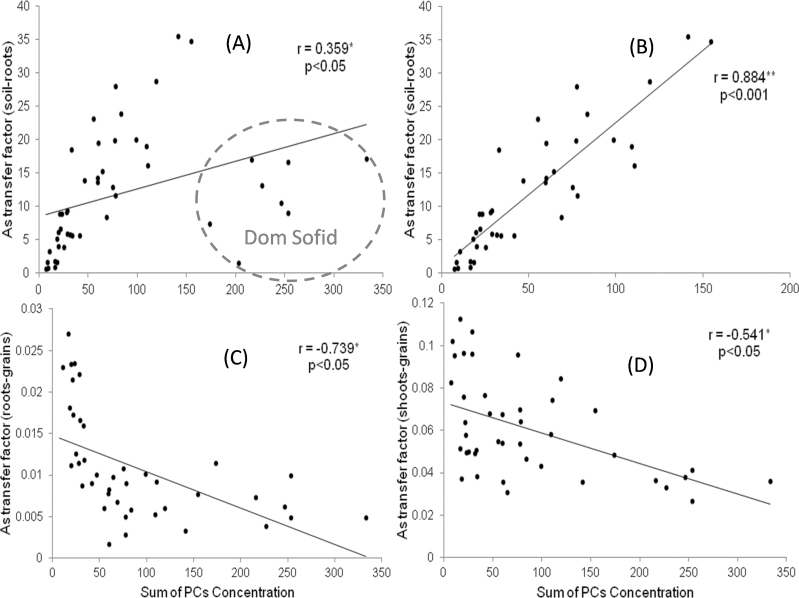
Scatter plots of PCs (sum of PCs concentration, SoP, free PCs+PCs As) on As transfer factor (TF) in all the cultivars studied: A (*n*=48), TF_soil–root_ versus SoP; B (*n*=40), TF_soil–root_ versus SoP with Dom Sofid removed; C (*n*=42), TF_roots–grain_ versus SoP (all cultivars); and D (*n*=42), TF_shoot–grain_ versus SoP (all cultivars).

TF_soil–root_ were weakly correlated with the sum of non-complexed PCs (*r*=0.359, *P* <0.05), whereas TF_root–grain_ and TF_shoot–grain_ showed strong negative correlations (*r*= –0.739 and –0.541, respectively, *P* <0.05). Significant positive correlations were observed between o-As (MMA and DMA) in grains and GSSG and non-complexed PCs in roots of high exposure samples (Table 2). Concentrations of i-As in grains versus PCs in roots (non-complexed and complexed) showed a negative correlation whereas i-As levels in grains were positively correlated with GSH in roots. Grain t-As correlated negatively with As–PC_4_ and As–Glu–PC_3_. GSH did not correlate with PCs. GSSG correlated significantly with low molecular weight PCs but otherwise only weakly with DMA and As–DesGly–PC_3_ (Table 2; for molecular weights see Supplementary Table S3 available at *JXB* online). Correlations were observed between TF_root–grain_ or TF_root–shoot_ and t-As in grains and some As–PCs (Table 2). Finally, a correlation was observed between TF_root–grain_ and the sum of PCs further correlations between all PCs (free PCs versus free PC and As–PCs; Table 2; Fig. 5).

**Table 2.** Correlation between t-As, DMA, MMA, and i-As of grains and GSH, GSSG, and As–PCs of roots and transfer factors (TF) from high exposure (*n*=24)

**Analytes**	SoP	**t-As**	DMA	**MMA**	i-As	**GSH**	GSSG	**OHMe **PC**_**2**_**	PC_2_	**DesGly PC2**	PC_2_ OXI	**Iso-PC**_**2**_ **Glu**	OHMe-PC3As	**PC**_**3**_	PC_3_As	**Iso-PC**_**3**_ **GluAs**	DesGly PC_3_As	**PC**_**4**_ **As**	Iso-PC_3_ Glu	**TFRS**
**t-As**	**0.174**																			
DMA	0.699**	0.643^**^																		
**MMA**	0.713**	**0.570** ^**^	**0.858** ^**^																	
i-As	–0.057	0.726^**^	0.317	0.302																
**GSH**	**0.020**	**0.170**	**0.214**	**0.105**	**0.442** ^*^															
GSSG	0.749**	0.041	0.417^*^	0.403	–0.252	0.086														
**OHMe PC**_**2**_	0.731**	**–0.026**	**0.314**	**0.292**	**–0.262**	**0.257**	**0.761** ^**^													
PC_2_ Red	0.809**	0.160	0.508^*^	0.483^*^	–0.178	0.049	0.719^**^	0.712^**^												
**DesGly**	0.856**	**0.139**	**0.569** ^**^	**0.491** ^*^	**–0.214**	**0.293**	**0.699** ^**^	**0.718** ^**^	**0.775** ^**^											
**PC**_**2**_																				
PC_2_ Oxi	0.800**	0.116	0.540^**^	0.486^*^	–0.317	0.008	0.797^**^	0.757^**^	0.902^**^	0.891^**^										
**Iso-PC**_**2**_	0.812**	**0.066**	**0.517** ^*^	**0.468** ^*^	**–0.302**	**0.042**	**0.800** ^**^	**0.691** ^**^	**0.794** ^**^	**0.902** ^**^	**0.921** ^**^									
**Glu**																				
OHMe	0.455*	–0.288	0.210	0.224	–0.161	–0.152	0.275	0.240	0.253	0.114	0.170	0.292								
PC_3_As																				
**PC**_**3**_	0.821**	**0.280**	**0.625** ^**^	**0.585** ^**^	**0.050**	**0.238**	**0.634** ^**^	**0.536** ^**^	**0.799** ^**^	**0.743** ^**^	**0.781** ^**^	**0.733** ^**^	**0.146**							
PC_3_As	0.555**	–0.322	0.238	0.296	–0.184	–0.083	0.405	0.294	0.345	0.220	0.246	0.378	0.928^**^	0.332						
**Iso-PC**_**3**_	0.643**	–0.425*	**0.130**	**0.386**	**–0.392**	**–0.162**	**0.416**	**0.289**	**0.310**	**0.139**	**0.289**	**0.440**	**0.776** ^**^	**0.153**	**0.756** ^**^					
**GluAs**																				
DesGly	0.668**	–0.185	0.283	0.458^*^	–0.241	–0.221	0.500^*^	0.321	0.432^*^	0.418^*^	0.419^*^	0.504^*^	0.763^**^	0.326	0.791^**^	0.741^**^				
PC_3_As																				
**PC**_**4**_ **As**	0.575**	**–0.485** ^*^	**0.111**	**0.225**	**–0.244**	**–0.210**	**0.292**	**0.155**	**0.284**	**0.168**	**0.229**	**0.321**	**0.853** ^**^	**0.311**	**0.910** ^**^	**0.756** ^**^	**0.833** ^**^			
Iso-PC_3_	0.767**	0.091	0.363	0.483^*^	–0.419	0.103	0.777^**^	0.853^**^	0.743^**^	0.814^**^	0.819^**^	0.782^**^	0.118	0.824^**^	0.238	0.319	0.329	0.052		
Glu																				
**TFRS**	**–0.297**	**0.244**	**–0.177**	**–0.086**	**0.186**	**–0.326**	**–0.027**	**0.052**	**–0.097**	**–0.222**	**–0.018**	**–0.043**	**–0.268**	**–0.015**	**–0.277**	**–0.301**	**–0.178**	**–0.159**	**0.262**	
TFRG	0.547**	0.468^*^	–0.053	–0.079	0.444^*^	–0.184	–0.241	–0.221	–0.324	–0.410^*^	–0.277	–0.296	–0.402	–0.204	–0.440^*^	–0.445*	–0.451^*^	–0.384	–0.118	0.842^**^

t-As, Total arsenic; DMA,: dimethyl-arsenic; MMA, monomethyl-arsenic; i-As, inorganic arsenic; SoP, sum of PCs concentrations; TF, transfer factor; TFRS, TF roots–shoots; TFRG, TF roots–grains; *P <0.05; **P <0.01.

## Discussion

### Total As in rice and agronomic parameters

According to the literature (Norton *et al.*, 2012; Tripathi *et al.*, 2012) different genotypes of rice cultivated under the same conditions exhibit different As uptake. Here, Italica Carolina and Lemont were the highest and the lowest grain As accumulators, respectively. The low grain t-As of Lemont matches data reported by Norton *et al.* (2009*a*, *b*; 2012) where Lemont was identified as a low As cultivar in multiple field experiments. The cultivars which accumulated more As in roots were those that had a higher As uptake and, consequently, transported more As to aerial parts (Fig. 1). Finally, the t-As in roots of Lemont shows that the iron plaques (Chen *et al.*, 2005) were effectively removed by applying the washing method, since no statistical difference was observed between low and high exposure levels (Fig. 1).

Arsenic had a strong negative influence on plant development as was clearly shown by the negative correlations with agronomic parameters and t-As (see Supplementary Tables S1 and S2 available at *JXB* online), confirming previous studies (Carbonell-Barrachina *et al.*, 1995,^1998^; Knauer *et al.*, 1999; Zhang *et al.*, 2002, Shri *et al.*, 2009). Italica Carolina appeared to be the most sensitive to As and YRL-1 the least sensitive cultivar.

### Thiol compounds and As–PC complexes in roots

Exposure to elevated As levels led to the increased formation of As–PC complexes compared with that at low exposure (Table 1). Bluemlein *et al.* (2008) showed that the same proportion of As complexed to biothiols was determined in plant extracts using the low temperature extraction, followed by the online HPLC-ICPMS/ESI-MS detection, as determined using direct speciation without sample preparation using XANES/EXAFS. The low-temperature extraction and the online separation/detection limit the *de novo* synthesis of As–PCs and their degradation during extraction and chromatographic separation (Bluemlein *et al.*, 2009). However, this was only shown for the plant *Thunbergia alata* and not for rice plants. This needs to be considered when the occurrence of the As–PC complexes is discussed.

In this regard, all cultivars contained PCs at low As exposure, but only Dom Sofid and Kitrana 508 contained As bound to PCs under these conditions. Both cultivars contained, on average, the same amount of thiols in the form of GSH as in the form of PCs at low As exposure whereas, in other cultivars, the amount of thiols from GSH were higher than that present as PCs. Exposure to elevated As levels led to increased PCs’ production. All cultivars, except Dom Sofid and Kitrana 508, increased their total PCs concentrations by at least a factor of three (Dom Sofid and Kitrana 508 by about 2) upon exposure to elevated As levels.

Some PCs produced by rice in general increased in the cultivars during exposure to high As levels. These were, in most cases, bound up in the form of As–PCs complexes and were not present as free PCs. Only the amount of free PC_2_ and DesGly–PC_2_ increased in some instances with elevated As. Tripathi *et al.* (2012) also found that GSH and GSSG concentrations varied according to As concentration and the cultivar (genotype) studied, especially in susceptible ones. The authors found that susceptible rice cultivar IET-4786 showed more pronounced oxidative stress compared with tolerant Triguna. They attributed this tolerance to the activity of enzymes such as serine acetyl transferase, cysteine synthase, γ-glutamyl cysteine synthase, arsenate reductase, PC synthase, and others.

As reported by Tripathi *et al.* (2012) As translocation depends on the cultivar sensitivity and the synthesis of PCs, which is non-dose-dependent. The authors stated that the synthesis of PCs increase with As^V^ concentration. However, above a certain concentration of As, there is a reduction in the synthesis of PCs. As previously observed, the present study indicated that a specific cultivar with different As TFs showed singular sensitivity to As. However, the total concentration of PCs did not give an indication of TFs.

### Inorganic and methylated As in grains

Total As levels in grains were dose- and cultivar-dependent (Figs 3, 5). Despite the fact that the soil originally contained predominantly i-As and was spiked with i-As, high amounts of methylated As-species were found in grains. This was most likely the result of soil bacteria, especially As-methylating ones, thriving under flooded culture conditions. It has been shown (Raab *et al.*, 2007b) that methylated As (especially DMA) is better translocated to aerial parts of rice than i-As, potentially due to (i) a lower ability to bind to PCs and be sequestered into root vacuoles and (ii) efficient transporters as shown by Li *et al.* (2009). The increased As levels in grains of most cultivars, except Lemont and YRL-1, at elevated As levels resulted from increased amounts of DMA and not of i-As.

### Arsenic translocation in rice cultivars

The transfer factor (TF) is defined as the long-range transport of a specific compound from one part into another under specific condition of cultivation (Carbonell *et al.*, 1998; Raab *et al.*, 2007b). TFs vary considerably between plant species and within cultivars of a single species (Zhao *et al.*, 2010). The most important TF of rice with regard to human nutrition is the TF_soil–grain_ (Fig. 2D). In these experiments it varied between 0.05 and 0.22 with Italica Carolina having the highest TF_soil–grain_ at low exposure. TF_soil–shoot_ values (Fig. 2F) found here for rice were similar to those found by Williams *et al.* (2007), being about 50 times higher than reported for wheat and barley. Comparison of the different exposure levels showed that TF_soil–root_ increased with the amount of As present in soil for all cultivars, but Lemont (Fig. 2A). By contrast, TF_roots–shoot_ decreased in all cultivars with increasing soil As content, except Lemont. Similarly, all other TFs (soil–grain, root–grain, and soil–shoot) either remained constant or decreased with elevated As levels in soil.

Considerable differences in TF factors were identified between cultivars. Kitrana 508 showed the highest As transference from soil to roots, but TF into shoots and grains was lower than for YRL-1 and Lemont. The mean roots to shoots/grains and shoots to grains TFs to low exposure level for YRL-1 (Fig. 2B, C, E) were higher than the TFs for the other cultivars, showing a predilection for As transfer from roots to shoots and roots to grains. However, TF_shoot–grain_ does not seem to be a good parameter to evaluate since no statistical differences were observed between low and high exposure samples (Fig. 2C).

### Role of thiol compounds and As–PCs on As transfer into grains

**Fig. 6.**
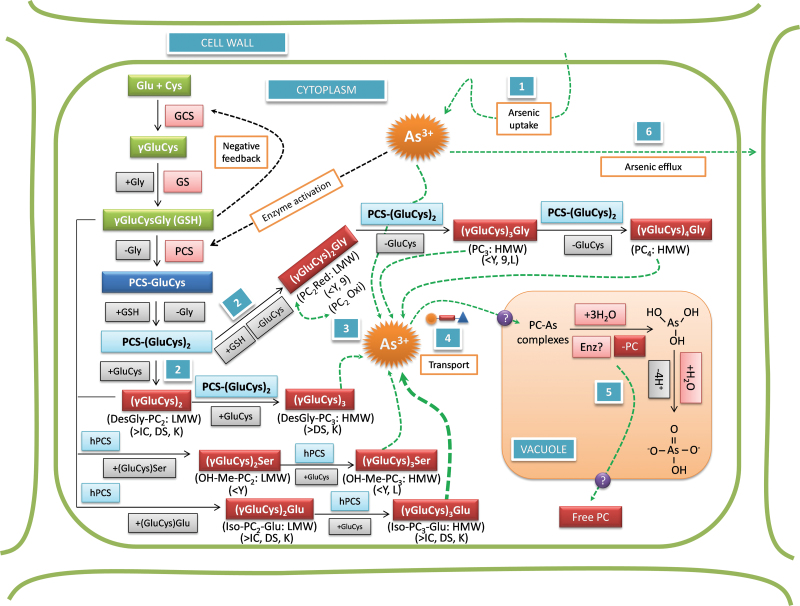
Probable pathways of phytochelatins (PCs) biosynthesis in roots of rice: data from literature, correlation between the PCs found here and the PCs related to the cultivars studied. Cultivars: L, Lemont; DS, Dom Sofid; IC, Italica Carolina; Y, YRL-1; 9, 9524; K, Kitrana 508. LMW, low molecular weight; HMW, high molecular weight; PCS, PC synthase; GCS, γ-glutamylcysteine synthetase; GS, glutathione synthetase; hPCS, homo-PC synthases; 1, As uptake by roots; 2, production of LMW PCs; 3, As–PCs complexes (As^III^+2PC_2_, As^III^+PC_3_, for example) stored in the vacuole; 4, transport to vacuoles; 5, release of PCs; 6, efflux of As up-taken; ?, unknown information; >, high concentration; <, low concentration; thicker dotted line, PC highly effective. Scheme elaborated according to descriptions from Klapheck *et al.* (1994); Cobbett (2000); Chassaigne *et al.* (2001); Cobbett and Goldsbrough (2002); Oven *et al.* (2002); Meharg and Hartley-Whitacker (2002); Rey *et al.* (2004); Vatamaniuk *et al.* (2004); Song *et al.* (2010); and Wood and Feldmann (2012).

To understand the correlations between PCs it is necessary to study the biosynthesis of those compounds. Initially, the enzyme γ-glutamylcysteine synthetase (GCS) produces γ-GluCys and glutathione synthetase (GS) completes the synthesis of GSH by adding Gly to the structure, producing γ-GluCysGly (Fig. 6). GSH, by negative feedback, controls the GCS activity (Cobbett, 2000; Cobbett and Goldsbrough, 2002). The synthesis of peptides (γ-GluCys)_n_Gly in plants is stimulated by toxic elements (such as As and Cd) being catalysed by the constitutive enzyme PC synthase (PCS), which transfers the glutamyl-cysteine fraction (γ-GluCys) from GSH to an acceptor molecule, GSH or peptide (γ-GluCys)_n_Gly, producing (γ-GluCys)_n+1_Gly, the PCs (Fig. 6). In this step the highest producer is Dom Sofid, followed by Kitrana 508 and Italica Carolina and, interestingly, the lowest are YRL-1 and 9524, cultivars that showed increased i-As in grains to high exposure level (Fig. 3; Table 1). PCs with no Gly at the C-terminus are named DesGly–PC. DesGly–PC_3_ was found here at high concentrations in Dom Sofid and Kitrana 508 (Fig. 6; Table 1). Different families and orders of plants may have PCs with different amino acids. For example, the order Fabales (monocotyledonous) produces (γ-GluCys)_n_Ala when exposed to Cd. The family Poaceae (grasses such as wheat, rice) can produce several iso-PCs such as (γ-GluCys)_n_Glu, (γ-GluCys)_n_Gly, (γ-GluCys)_n_Ser (also named hydroxymethyl-PC), and (γ-GluCys)_n_ (Klapheck *et al.*, 1994; Cobbett and Goldsbrough, 2002; Vatamaniuk *et al.*, 2004). These PCs are the product of homo-PC synthases, which can use γ-GluCys units as substrate from GSH, hGSH (γ-GluCys-β-Ala), PCs, and DesGly–PCs (Oven *et al.*, 2002), all found in the present study. Ser–PCs and Glu–PCs were more prevalent in Dom Sofid, Italica Carolina, and Kitrana 508, cultivars that did not show statistical difference for i-As in grains to low and high exposure levels (Fig. 3).

Figure 6 shows the biosynthesis pathways described in the literature and the relation with the data found in this study. Lemont probably has a special mechanism where small amounts of As are transported into the roots (step 1) or has a high As efflux (step 6). That would explain the low biosynthesis of PCs in Lemont, similar to YRL-1 and 9524 (low concentration of PCs and high i-As in grains) (Table 1). On the one hand, these last two cultivars presented low concentrations of DesGly–PC_2_, Iso–PC_2_–Glu, PC_2_oxi, PC_3_, and Iso–PC_3_–Glu (Table 1). In particular, DesGly–PC_2_ did not change its levels in these cultivars after high exposure (Table 1). On the other hand, Kitrana 508, Dom Sofid, and Italica Carolina substantially increased their levels of DesGly–PC_2_ (Table 1). This culminated in the high production of other PCs (Iso–PC_3_–Glu and Iso–PC_2_–Glu).

Regarding the reduction of 1.6 times in the concentration of grains i-As in Italica Carolina from low to high exposure levels (Fig. 3), we observed that Iso–PC_3_–Glu was highly expressed after exposure (Table 1), even in Kitrana 508 and Dom Sofid, comprising all the low i-As grain accumulators. Furthermore, Italica Carolina presented low levels of As–Iso–PC_3_–Glu, showing the importance of steps 4 and 5 (Fig. 6). Regarding step 4, Song *et al.* (2010) described that two ABCC-type vacuolar PC-transporters (AtABCC1 and AtABCC2) have an important role on As tolerance in *Arabidopsis* since they were associated with vacuolar As accumulation (As–PC_2_) and, interestingly, to PC biosynthesis. In this regard, Ueno *et al.* (2010) reported a gene (*OsHMA3*), responsible for low Cd accumulation in rice which encodes a P_1B_-type ATPase family transporter, sequestrating Cd into the root vacuoles. Further, step 5 is associated with the release of PC in the vacuole and return to the cytoplasm. This recycling probably has an important influence on the translocation, i.e. trapping of i-As in the roots, than the production or PCs’ levels itself. This is evidenced by comparing the levels of Kitrana 508’s PCs and Dom Sofid (Table 1).

The correlation between TF_soil–root_ and the sum of PCs concentration showed a weak positive statistical significance. As seen in Fig. 4A, Dom Sofid showed a very different relationship between TF_soil–root_ compared to other cultivars because it produced significantly more PCs than the other cultivars (Table 1). Removing Dom Sofid, the correlation becomes very strong (Fig. 4B). Total As in roots was positively correlated with the sum of PCs in all cultivars, but As transfer from roots and/or shoots to grains was significantly reduced by increased PCs’ production (Fig. 4C, D). This shows that PCs provide an important mechanism to decrease the concentration of i-As in grains and suggests that PC bound As was less likely to be transported into shoots and grains. Similar observations were made by Liu *et al.* (2010) in *Arabidopsis thaliana*. By contrast with the negative correlation between i-As in grains and PCs in roots, the GSH concentration was positively correlated with the amount of i-As in grains. GSH participates not only in production of PCs (Cobbett and Goldsbrough, 2002) and in antioxidant defence mechanisms, but also in the conversion of As^V^ in As^III^ (Raab *et al.*, 2004; Bleeker *et al.*, 2006; Duan *et al.*, 2011), which might be the reasons for the positive correlation.

No correlation was found between GSH and any of the individual PCs quantified, but individual PCs concentrations were correlated with the amount of GSSG. Strong correlations existed between individual PCs especially within a family, supporting the fact that chain elongation is taking place (Fig. 6; Table 2). The correlations between the sum of PCs and individual PC concentrations were stronger for free PCs than for those bound to As. The correlations between DesGly–PC_2_ and other PCs may indicate that, similar to microorganisms and yeast (Klapheck *et al.*, 1994; Cobbett and Goldsbrough, 2002; Zhao *et al.*, 2010), rice roots cells can also polymerize γ-GluCys directly with or without the addition of Gly or other amino acids. The cultivars showed individual differences not only in PC production but, potentially more importantly, in the different PC-families produced in response to As. Italica Carolina responded to high As exposure especially with increases of PC_2oxi_ and PC_3_ besides DesGly–PC_2_. This PC was also strongly elevated by As in cultivars 9524 and Kitrana 508. Both of these cultivars also increase the amount of Glu–PC_2_ while 9524 had lower Glu–PC_3_ than other cultivars. In these cultivars the PCs were not only positively correlated with the As concentration in roots but also with reduced i-As transfer to grains. YRL-1 did not produce many different PCs and was generally less sensitive to As compared with Italica Carolina. Lemont, despite its low total As uptake into the roots (independent of exposure), produced PCs in response to As which appear to reduce As transfer to grains even further.

Some cultivars, especially those which produced significant amounts of PCs upon exposure to elevate i-As, might also respond to exposure to other soil contaminants such as Cd, which stimulates PC synthesis. Hence, a co-exposure of As and Cd will probably influence the TFs of i-As. This might explain the site to site variation of As accumulation in rice grains observed by Norton *et al.* (2009*a*, *b*, 2012). Those authors found that Lemont, as well as CT9993-5-10-1-M, Azucena, and Teqing cultivars, was the lowest in grain As concentration, with potential use on breeding seeds. In six different fields in Bangladesh, China, and India, Lemont was one of the lowest As grain accumulator. This cannot be explained by the high amount of PCs or thiols production, but must be the result of another mechanism. The agreement between that study and this suggests Lemont is indeed a promising cultivar for breeding low grain As, and the results here suggest that its low TF_soil–shoot_ is the main reason for a low grain i-As. Exactly why its TF_soil–shoot_ is low is still not clear.

## Conclusion

In general, t-As in grains reflected the concentration in roots. Cultivars with higher As uptake (all species) from soil translocate As more efficiently to the grains. Elevated As concentrations in roots, shoots, and grains negatively affected plant development and, consequently, grain yield, especially in cultivar Italica Carolina.

Cultivars with higher As-uptake into roots, such as Dom Sofid and Italica Carolina, produced higher concentrations of PCs than other cultivars. All rice cultivars produced a range of different PCs belonging to the Des–Gly–PC_n_, PC_n_, Ser–PC_n_, and Glu–PC_n_ families with *n* varying from 2 to 4. Correlations between different PCs proved that chain elongation by one γ-Glu-Cys unit is used by rice for the production of PCs.

With the exception of cultivar Lemont, t-As in grains increased with increasing As exposure. Speciation of As in grains revealed that elevated As exposure resulted in increased concentration of DMA and also MMA in grains. PCs’ production and the formation of As–PC complexes in roots reduced the transfer of i-As into shoots and grains, but had no influence on DMA and MMA transfer.

Transfer factors for As from the soil/root to grains were lower when the plants were exposed to elevated As levels due to the stimulation of more extensive production of PCs and, consequently, reduced As transport to aerial parts of the plant. Results also showed that the uptake of methylated As species is different between cultivars, probably due to soil microbial methylation of As and As efflux from roots.

This study showed clearly that As-uptake and transfer into grains strongly depends on rice cultivar and bioavailability of As in soil. Some cultivars like Dom Sofid, Kitrana 508, and Italica Carolina are better able to mobilize As from soil than others, but efficiently trap the most toxic form, As^III^, in the roots. Knowing the characteristics of each genotype (cultivar) and concentration (availability) of arsenic in soil, different cultivars can be used to prevent or reduce the arsenic contamination of grains and, consequently, the toxicological/nutritional risk, especially of i-As. Finally, this research suggests that modifications of PCs in plant roots will have implications on grain As levels and more research is need to investigate if this can be exploited in crop improvement targeting the reduction of As exposure risk.

## Supplementary data

Supplementary data can be found at *JXB* online.

Supplementary Table S1. Description of agronomic parameters.

Supplementary Table S2. Correlations between agronomic parameters and t-As (*n*=48, low and high exposure levels).

Supplementary Table S3. Theoretical, experimental, and accurate masses (Δm), retention time (RT), and molecular formula of GSH, free PCs, and PCs-As identified and quantified in the present study.

Supplementary Data
