# Supplementary material for: Identification and quantification of phytochelatins in roots of rice to long-term exposure: evidence of individual role on arsenic accumulation and translocation
Source: J Exp Bot. 2014 Mar 5;65(6):1467–79. doi: 10.1093/jxb/eru018 (PMC3967088; doi:10.1093/jxb/eru018)
Supplement: Supplementary Data [file supp_65_6_1467__index.html]

Identification and quantification of phytochelatins in roots of rice to long-term exposure: evidence of individual role on arsenic accumulation and translocation — Identification and quantification of phytochelatins in roots of rice to long-term exposure: evidence of individual role on arsenic accumulation and translocation — Supplementary Data 

# Identification and quantification of phytochelatins in roots of rice to long-term exposure: evidence of individual role on arsenic accumulation and translocation

## Supplementary Data

Data files

**Files in this Data Supplement:**

- Supplementary Data - Supplementary Data
