# Supplementary material for: Identification and quantification of phytochelatins in roots of rice to long-term exposure: evidence of individual role on arsenic accumulation and translocation
Source: J Exp Bot. 2014 Mar 5;65(6):1467–79. doi: 10.1093/jxb/eru018 (PMC3967088; doi:10.1093/jxb/eru018)
Supplement: Supplementary Data [file supp_eru018_jexbot108928_file001.pdf]

Supporting Information for:

Identification and quantification of phytochelatins in roots of rice to long-term exposure: evidence of individual role on arsenic accumulation

Bruno Lemos Batista[a,e], Meher Nigar[b], Adrien Mestrot[c], Bruno Alves Rocha[d], Fernando Barbosa Júnior[e], Adam H. Price[b], Andrea Raab[f], Jörg Feldmann[f]*

[a]Centro de Ciências Naturais e Humanas, Universidade Federal do ABC, Bloco B, Av. dos Estados 5001, Santo André (SP), Brazil.

[b]Institute of Biological and Environmental Sciences, University of Aberdeen, Cruickshank Building, St. Machar Drive, Aberdeen AB24 3UU, UK.

[c]Soil Science Group, Institut of Geography, University of Bern, Hallerstrasse 12, 3012, Bern, Switzerland.

[d] Departamento de Química, Faculdade de Filosofia Ciências e Letras de Ribeirão Preto, Universidade de São Paulo, Avenida Bandeirantes, 3900, Ribeirão Preto (SP), Brazil.

[e]Faculdade de Ciências Farmacêuticas de Ribeirão Preto, Universidade de São Paulo, Bloco A, Av. do Café s/n, Ribeirão Preto (SP), Brazil.

[f]School of Engineering and Physical Science, University of Aberdeen, MestonWalk, Aberdeen, Scotland, AB24 3UE, UK.

* Corresponding author: e-mail: j.feldmann@abdn.ac.uk

Supporting Information Table S1. Description of agronomic parameters. Results expressed as mean or mean ± standard deviation (n=4).

**Rice cultivar**	**Exposure level**	**Abbreviation**	**F(days)**	**PH(cm)**	**SD(mm)**	**GY(g)**	**SS(mm)**	**NT**	**PM(g)**
Italica Carolina	*Low*	IC(L)	51	101.5*	22.8±2.5	9.59*	7.77±0.78*	5	102.8*
	High	IC(H)	51	89.6	17.8±4.4	4.34	6.71±0.65	4	90.6
Dom Sofid	*Low*	DS(L)	87	137.5	19.0±3.1	7.18*	8.04±0.43	4	138.4
	High	DS(H)	88	128.5	17.7±6.9	5.40	8.05±0.65	4	129.4
9524	*Low*	9(L)	91	140.5	23.1±2.5	10.64	7.13±0.51*	4	141.4
	High	9(H)	94	133.8	19.7±2.6	8.68	6.73±0.44	4	134.6
Kitrana 508	*Low*	K(L)	94	155.3	17.8±2.2	6.99*	7.49±0.16*	4	156.1
	High	K(H)	94	150.5	16.1±1.5	5.80	7.00±0.24	3	151.3
YRL-1	*Low*	Y(L)	91	115.8	24.1±4.8	11.81	7.64±0.45	5	116.9
	High	Y(H)	91	110.5	23.7±6.2	10.17	7.37±0.54	5	111.8
Lemont	*Low*	L(L)	102	76.9	27.1±2.0*	9.46	7.34±0.45	4	77.9
	High	L(H)	107	83.4	21.3±2.9	7.88	7.18±0.44	3	84.2

PH: plant height; **PM**: plant mass (biomass mean of roots+shoots+grains); **F**: time to flowering (mean of 4 replicates); **SD:** stems diameter; **GY:** grain yield (mean of biomass of all panicles produced by the plant); **SL:** seed length; **NT:** number of tillers; *****: significant statistical differences between low and high exposure level (P<0.05).

Supporting Information Table S2. Correlations between agronomic parameters and t-As (n= 48, low and high exposure levels).

Variables	Flowering (days)	Plant height (cm)	Grains weight (g)	Shoots diameter (mm)	Roots weight (g)	Shoots weight (g)	All plant weight (g)	[As] roots (µg kg^{-1})	[As] shoots (µg kg^{-1})	[As] grains (µg kg^{-1})
Plant height (cm)	.758**									
Grains weight (g)	.156	.090								
Shoots' diameter (mm)	.559**	.401*	.269							
Roots weight (g)	.713**	.730**	.484**	.458**						
Shoots weight (g)	.649**	.619**	.576**	.618**	.813**					
All plant weight (g)	.720**	.749**	.517**	.494**	.989**	.865**				
[As] roots (µg kg^{-1})	-.118	-.166	-.775**	-.339	-.366*	-.602**	-.421*			
[As] shoots (µg kg^{-1})	-.353*	-.259	-.631**	-.369*	-.541**	-.685**	-.581**	.740**		
[As] grains (µg kg^{-1})	-.191	-.286	-.552**	-.330	-.342	-.458**	-.372*	.685**	.739**	
[As] soil (µg kg^{-1})	-.066	-.282	-.621**	-.059	-.322	-.336	-.369*	.575**	.342	.355*

[As]: arsenic concentration; *: P<0.05; **: P<0.01.

Supporting Information Table 3. Theoretical, experimental and accurate masses (Δm), retention time (RT) and molecular formula of GSH, free PCs and PCs-As identified and quantified in the present study. Values presented as mean ± standard deviation (n=48), . NI: non ionized.

**Compound**	**$[GSH+H]^+$**	**$[As\text{-}OH\text{-}Me\text{-}PC_3+H]^+$**	**$[GSSG+H]^+$**	**$[PC_3+H]^+$**
Theoretical m/z	308.0916	874.1039	613.1598	772.1951
Experimental m/z	308.0907±0.0002	874.1022±0.0011	613.1584±0.0004	772.1937±0.0009
Δm (ppm)	3.02±0.90	1.86±1.32	2.06±1.09	1.82±1.17
RT (min)	3.93±0.11	17.95±0.16	7.47±0.18	18.49±0.25
Molecular formula (NI)	$C_{10}H_{17}O_6N_3S$	$C_{27}H_{40}O_{15}N_7S_3As$	$C_{20}H_{32}O_{12}N_6S_2$	$C_{26}H_{41}O_{14}N_7S_3$
**Compound**	**$[OH\text{-}Me\text{-}PC_2+H]^+$**	**$[As\text{-}PC_3+H]^+$**	**$[PC_2$ Reduced$+H]^+$**	**$[As\text{-}Iso\text{-}PC_3\text{-}Glu+H]^+$**
Theoretical m/z	570.1539	844.0932	540.1434	916.1145
Experimental m/z	570.1527±0.0003	844.0918±0.0012	540.1422±0.0003	916.1135±0.0015
Δm (ppm)	2.22±0.67	1.67±1.45	2.08±0.68	1.09±1.61
RT (min)	11.22±0.27	18.59±0.16	11.83±0.27	19.04±0.87
Molecular formula (NI)	$C_{19}H_{31}O_{11}N_5S_2$	$C_{26}H_{38}O_{14}N_7S_3As$	$C_{18}H_{29}O_{10}N_5S_2$	$C_{29}H_{42}O_{16}N_7S_3As$
**Compound**	**$[DesGly\text{-}PC_2+H]^+$**	**$[As\text{-}DesGly\text{-}PC_3+H]^+$**	**$[PC_2$ oxidized$+H]^+$**	**$[As\text{-}PC_4+H]^+$**
Theoretical m/z	483.1219	787.0718	538.1278	1076.1451
Experimental m/z	483.1205±0.0003	787.0704±0.0005	538.1266±0.0003	1076.1438±0.0016
Δm (ppm)	2.95±0.80	1.83±0.71	2.11±0.68	1.28±1.35
RT (min)	12.28±0.33	19.48±0.33	12.61±0.22	23.42±0.34
Molecular formula (NI)	$C_{16}H_{26}O_9N_4S_2$	$C_{24}H_{35}O_{13}N_6S_3As$	$C_{18}H_{27}O_{10}N_5S_2$	$C_{34}H_{50}O_{18}N_9S_4As$
**Compound**	**$[Iso\text{-}PC_2\text{-}Glu+H]^+$**		**$[Iso\text{-}PC_3\text{-}Glu+H]^+$**	
Theoretical m/z	612.1645		844.2163	
Experimental m/z	612.1634±0.0006		844.2163±0.0021	
Δm (ppm)	1.87±0.99		0.01±2.42	
RT (min)	14.30±0.25		20.75±0.60	
Molecular formula (NI)	$C_{21}H_{33}O_{12}N_5S_2$		$C_{29}H_{45}O_{16}N_7S_3$	
